# Dedicated call center (SOS-HAE) for hereditary angioedema attacks: study protocol for a randomised controlled trial

**DOI:** 10.1186/s13063-016-1350-0

**Published:** 2016-04-30

**Authors:** Nicolas Javaud, Olivier Fain, Isabelle Durand-Zaleski, David Launay, Laurence Bouillet, Anne Gompel, Alain Sobel, Maguy Woimant, Hasina Rabetrano, Tomislav Petrovic, Frédéric Lapostolle, Isabelle Boccon-Gibod, Paul-Georges Reuter, Philippe Bertrand, Brigitte Coppere, Bernard Floccard, Gisele Kanny, Ludovic Martin, Eric Vicaut, Frédéric Adnet

**Affiliations:** Service des Urgences, Hôpital Louis Mourier, Centre de Référence associé sur les angiœdèmes à kinines (CRéAk), Assistance Publique - Hôpitaux de Paris, Université Paris 7, 178 Rue des Renouillers, 92 700 Colombes, France; Service de Médecine Interne, DHUi2B, Centre de Référence associé sur les angiœdèmes à kinines (CRéAk), Assistance Publique - Hôpitaux de Paris, Hôpital Saint-Antoine, Université Paris 6, 75 012 Paris, France; URCEco Ile de France, Assistance Publique - Hôpitaux de Paris, Hôpital de l’Hôtel-Dieu, Université Paris 12, 75 004 Paris, France; Service de Médecine Interne, Centre de Référence associé sur les angiœdèmes à kinines (CRéAk), Université de Lille, CHRU de Lille, 59037 Lille, Cedex France; Service de Médecine Interne, Centre de Référence associé sur les angiœdèmes à kinines (CRéAk), CHU de Grenoble, 38043 Grenoble, France; Département d’Endocrinologie Gynécologique, Centre de Référence associé sur les angiœdèmes à kinines (CRéAk), Assistance Publique - Hôpitaux de Paris, Hôpital Port Royal, Université Paris 5, 75001 Paris, France; T2i, Centre de Référence associé sur les angiœdèmes à kinines (CRéAk), Assistance Publique - Hôpitaux de Paris, Hôpital Hôtel Dieu, Université Paris 5, 75004 Paris, France; SAMU-SMUR 93, Assistance Publique - Hôpitaux de Paris, Hôpital Avicenne, Université Paris 13, 93 000 Bobigny, France; Service de Médecine Interne, et, 69 437, Lyon, Cedex France; Service de Réanimation, Centre de Référence associé sur les angiœdèmes à kinines (CRéAk), CHU Edouard Herriot, 69 437 Lyon, Cedex France; Service de Médecine Interne, Centre de Référence associé sur les angiœdèmes à kinines (CRéAk), CHU de Nancy, 54 035 Nancy, France; Service de Dermatologie, Centre de Référence associé sur les angiœdèmes à kinines (CRéAk), Université d’Angers, CHU d’Angers, 49 933 Angers, Cedex France; Unité de Recherche Clinique, Assistance Publique - Hôpitaux de Paris, Hôpital Fernand Widal, Université Paris 7, 75 010 Paris, France

**Keywords:** Hereditary angioedema, Emergency, Icatibant, Plasma-derived C1 inhibitor, Call centre

## Abstract

**Background:**

Despite the availability of guidelines for the specific treatment of hereditary angioedema (HAE) attacks, HAE morbidity and mortality rates remain substantial. HAE attacks are a major medical issue requiring specific treatment as well as a considerable socio-economic burden. We report a protocol designed to test whether a dedicated call centre is more effective than usual practice in the management of patients experiencing an HAE attack.

**Methods/design:**

This prospective, cluster-randomised, single-blind, parallel-group, multicentre trial evaluates the morbidity and consequent socio-economic costs of the management of patients experiencing an HAE attack by a dedicated call centre as compared to usual practice. The trial aims to recruit 200 patients. Patients in the intervention arm are provided with an SOS-HAE card with the call centre’s freephone number that they can access in the case of an attack. The centre’s mission is to provide recommended expert advice on early home treatment. The centre can route the call to a local emergency medical service with competency in HAE management or even arrange for the drugs needed for the specific treatment of an HAE attack to be sent to the emergency department of the local hospital. The primary outcome measure is the number of hospital admissions for an HAE attack. Each patient will be followed up every 2 months for 2 years. The study has been approved by the ethics committee (*Comité de Protection des Personnes d’Ile de France 10*; registration number: 2012-A00044-39; date of approval: 19 January 2012).

**Discussion:**

The SOS-HAE protocol has been designed to address the handling of attacks experienced by patients with HAE in the home. The proposed trial will determine whether the setting up of a dedicated call centre is more effective than usual practice in terms of reducing morbidity as given by the numbers of hospital admissions. The results are also anticipated to have important implications in terms of socio-economic costs for both healthcare services and patients.

**Trial registration:**

ClinicalTrials.gov NCT01679912.

## Background

Hereditary angioedema (HAE) is a rare disease. Worldwide prevalence ranges from 1/50,000 to 1/100,000 [1]. The form exhibiting C1 inhibitor (C1-INH) deficiency is an autosomal dominant disorder, although 25 % of cases are due to a spontaneous mutation in individuals with no family history of the disease [2, 3]. Patients with C1-INH deficiency may have type I or type II HAE [2, 3]. Patients with a family history of angioedema but with normal C1-INH levels may have a mutation of the gene encoding human coagulation factor XII (FXII-HAE) [2].

HAE attacks are characterised by recurrent subcutaneous swelling without itching lasting 48 to 72 hours [3, 4]. By far the most common sites of attacks are the extremities and abdomen, each accounting for nearly half of all attacks [5]. Abdominal attacks can cause severe pain and third-space fluid accumulation with resultant hypotension [5, 6]. Moreover, more than half of all HAE patients experience at least one laryngeal attack during their lifetime with the associated risk of asphyxiation [7]. Despite the availability of guidelines on specific treatments for HAE attacks, morbidity and mortality rates remain substantial [2, 3, 8].

The management of patients with HAE is shifting towards self-treatment of attacks at home with fewer visits to the hospital emergency department (ED) and fewer admissions [9, 10]. Even so, in a recent retrospective study of 193 HAE patients with 8 attacks/patient/year, approximately 11 % of patients visited the ED [11]. Moreover, according to a prospective observational study including 29 HAE patients, laryngeal and facial edemas are independent risk factors associated with hospital admission, and this is not avoided by early ED arrival [12]. A prospective study on the impact of measures, in particular specific home treatments, that might shorten the time from attack onset to first contact with a healthcare professional is required [12].

HAE attacks are not only an important medical issue but also a socio-economic issue [13]. Costs and hospital stays increase with attack severity [13]. A randomised study evaluating a telephone care-management strategy versus usual practice reported a significant decrease of average medical costs and hospital admissions in the telephone-coached group [14]. The aim of our multicentre, cluster-randomised, controlled trial is to determine whether a central dedicated call centre for HAE attacks that provides guidelines-based, standardised advice on early treatment would reduce the number of hospital admissions (morbidity) and thus the socio-economic impact of the disease.

## Methods/design

### Design and setting

The SOS-HAE trial is a prospective single-blind, two-arm, cluster-randomised, multicentre trial for patients with HAE attending a reference centre for bradykinin-mediated angioedema. Because the prevalence of HAE is low, the study will include only adult patients with an HAE diagnosis already confirmed by a specialist on the basis of patient history, functional and antigenic C1-INH levels and genetic data. Each investigator will select eligible patients (those with type I HAE, type II HAE or FXII-HAE) from amongst his/her reference centre’s customary patient population.

### Ethical aspects

The SOS-HAE study protocol and patient information sheets have been approved by the ethics committee and by the competent French authorities (*Comité de Protection des Personnes d’Ile de France 10; Hôpital Robert Ballanger*; registration number: 2012-A00044-39; date of approval: 19 January 2012).

Patients are informed orally and in writing (SOS-HAE information sheet) by the investigator and can refuse to participate. Written informed consent is required by French law, as the standard of care does not apply to both study arms.

### Participating centers

Eight French reference centres for bradykinin-mediated angioedema are participating in the trial. The centres were accredited in 2006 by the French Ministry of Health. Their mission is to improve access to diagnosis and therapy for patients with HAE. All eight centres have medical and paramedical teams with experience in the field of HAE, all implement a therapeutic education programme based on guidelines for systematic treatment of severe attacks and for prophylaxis of recurrent attacks, and all offer nurse-led sessions on self-administration of specific therapy for an attack [2, 3, 8, 15].

The freephone call centre (0800 111 001) is hosted by the French emergency medical service (EMS) of a Paris suburb (SAMU 93) which has the logistics capabilities needed to provide 24/7/365 expert medical advice over the telephone [16]. SAMU 93 has promoted appropriate care of HAE attacks by (1) posting placards in SAMU 93 to enhance awareness and (2) providing their emergency physicians with instruction sheets on recommended emergency management of HAE attacks and prophylaxis of recurrence [2].

In France, each local EMS is equipped with one or more mobile intensive care units (MICUs), which include a senior emergency physician, a nurse and an ambulance driver. MICUs provide rescue techniques and medical advice, known as advanced life support, throughout France [16]. The local EMS facilities of each participating reference centre have the required logistics capabilities for storage and around-the-clock delivery of appropriate specific agents (plasma-derived C1-INH and icatibant) to patients or healthcare organisations [17]. If the case is life-threatening, SOS-HAE will immediately contact SAMU headquarters.

### Study population

Eligible patients are adults (≥18 years) with HAE at one of the eight participating reference centres. Inclusion criteria are a documented diagnosis of HAE (type I, II or FXII-HAE) and signed informed consent. Exclusion criteria are pregnancy, recent history (≤1 month) of myocardial infarction or ischemic stroke and allergy to icatibant or plasma-derived C1-INH.

Withdrawal of the study: withdrawal of consent follow-up.

### Randomisation

The randomised units are the reference centres (clusters) with randomisation stratified according to the number of eligible HAE patients by reference centre. Using permuted blocks of 4, we randomly assigned reference centres (not patients) to the intervention or to usual practice (1:1 ratio).

A modified version of Zelen’s method involving a two-step informed consent process was used for randomisation and inclusion [18]. Eligible patients were first invited to participate in a cohort study but without being informed of the nature of the study arms. Patients who agreed to participate in the study signed the first consent form. This was followed by verification of eligibility (compliance with inclusion/exclusion criteria) and centre randomisation. The patients in each centre were then offered inclusion in the arm assigned to their centre but without being informed of the nature of the other arm. Patients who agreed to be included signed the second consent form. The patient inclusion day is study day zero (D0).

### Study arms

**Fig. 1:**
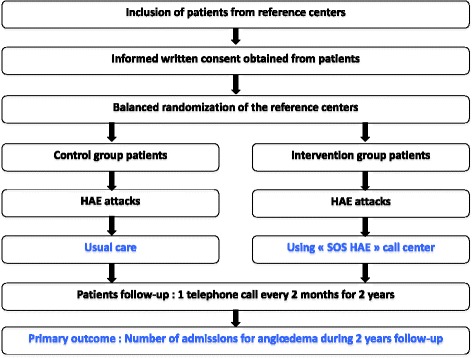
Study design

**Fig. 2:**
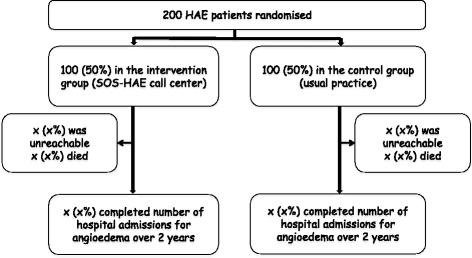
Study flowchart

The study protocol and randomisation arms are described in Fig. 1. The methodical management strategy of patients with HAE experiencing an HAE attack (SOS-HAE) was conceptualised after discussions of inclusion criteria and follow-up with a specialist in HAE. The protocol assesses the impact of the implementation of this strategy on number of hospital admissions in a group of patients rather than the actual efficacy of the treatment administered (Fig. 2)*.*

### Intervention arm

All patients in the intervention arm are given an SOS-HAE card indicating what to do in the case of an attack: *“On attack onset and before taking any drugs, call the freephone number 0800 111 001*”. The physicians receiving the call at the call centre (open 24/7/365) have received special training as well as instruction sheets on emergency HAE management according to attack severity (Table 1). They can prescribe home treatment or route the call to a local EMS team with competency in HAE management. They can also make arrangements for specific drugs to be sent to the ED of the patient’s local hospital.

**Table 1:** Treatment recommendations

Treatment	Recommendations
Vital distress	- Immediately and as soon as possible:
	- Administer icatibant (Firazyr®): 30 mg subcutaneously or
	- plasma-derived C1-INH (Bérinert®): 20 UI/kg intravenously
	- Switch the call to the local SAMU to send French EMS
	- Gain control of upper airway
Severe attacks	- Immediately and as soon as possible:
Laryngeal	- Administer icatibant (Firazyr®): 30 mg subcutaneously or
Facial	- plasma-derived C1-INH (Bérinert®): 20 UI/kg intravenously
Abdominal	- If treatments are unavailable at home, switch the call to the local SAMU to send an ambulance headed towards a hospital with specific treatments available or being able to get them by French EMS
	- Gain control of upper airway
Non-severe attacks (members, genitals)	- Tranexamic acid: 1 g/6 h except for patients who are breastfeeding or have thromboembolic pathology
Surveillance in all cases	Monitoring by phone 30 min, 1 h, 4 h, 12 h and 24 h after the beginning of the attack
	Advice to call back SOS-HAE call centre in case of secondary worsening

#### Usual practice arm

No changes are made to usual practice treatment, and patients are not given the SOS-HAE card.

### Data collection and follow-up

#### Medical data

HAE specialists and emergency physicians collect standardised clinical data including those presented in the most recent Hereditary Angioedema International Working Group (HAWK) guidelines [2]:

- On day zero (D0): Patient sex, age, type of HAE (type I, II or FXII-HAE), time since diagnosis, personal history of angioedema (ED visits during previous year, admissions to intensive care unit (ICU) and history of intubation and tracheotomy), ongoing long-term treatment and number of relatives with HAE
- At each attack and every 2 months for 2 years: Number of attacks and, for each attack: possible trigger of attack, day and time of onset of symptoms, day and time of call to SOS-HAE centre, edema site, home therapy (self-administered or caregiver) or hospital treatment, time treatment started and course of attack (onset of symptom relief and time of symptom resolution)
- Number of admissions/patient/year over 2 years, duration of admissions, number of admissions to ICU/patient/year over 2 years, number of ED visits/patient/year over 2 years, number of intubations, number of interventions by EMS and mortality

#### Health economics data

Call centre costs include freephone, training of EMS personnel and answering of calls (time spent and number of calls).

Patient care costs include consumption of hospital resources (admissions) and out-of-hospital resources (ED visits or emergency ambulance, drugs to treat attacks and their administration by physicians or nurses), working days lost and any remaining costs supported by patients.

#### Quality of life

The Short Form Health Survey (SF-36) score is measured after 1 year and after 2 years of follow-up.

### Trial coordination and implementation

Each medical and paramedical team in the eight participating reference centres and in the SOS-HAE call centre is trained in protocol implementation and data collection. The electronic case report form (eCRF) was developed using CleanWEB™ (a centralised, secure, interactive web response system edited by Telemedicine Technologies and accessible from each study centre). In accordance with French law, the eCRF and database were validated by the CCTIRS (*Comité consultatif sur le traitement de l’information en matière de recherche dans le domaine de la Santé -* Advisory Committee on Information Processing in Healthcare Research) and by the CNIL (*Commission Nationale de l’Informatique et des Libertés* - French Data Protection Authority).

### Blinding

Physicians and nurses could not be blinded to the intervention given its nature, but patients are blinded to the intervention by Zelen’s method (pre-randomisation consent). The single-blind procedure is partially counterbalanced by the objective nature of the primary outcome measure [19]. The analysis will be blinded to group allocation.

### Outcome measures

#### Primary outcome

The primary outcome is the number of admissions for angioedema attacks per patient per year over a 2-year period. The number of admissions for angioedema attacks is measured from the randomisation date until the end of follow-up or death. For patients discharged alive, information on the primary outcome will be collected by phoning the patients. All admission observation charts are collected and collated.

#### Secondary outcomes

Secondary outcomes are the number of admissions for a cause other than an angioedema attack per year over a 2-year period, mortality from an angioedema attack, mortality from another cause, number of ICU admissions per year, number of ED admissions per year, number of hospital stays, number of intubations per year, number of interventions by EMS, number of working days lost and their duration, costs of patient care and SF-36 score.

#### Definitions

An admission is defined as a hospital stay >12 hours as an inpatient. An ED visit is defined as a consultation in the ED without admission (stay ≤24 hours).

### Sample size calculation

The aim of this study is to demonstrate a difference in outcome between a methodical management strategy and usual practice. Our primary hypothesis is that an SOS-HAE call centre might benefit patients suffering from an angioedema attack. The sample size calculation is based on the primary outcome, i.e. on the number of admissions for angioedema attacks.

A recent study involving 193 patients with HAE in France reported a rate of approximately 8 attacks/year/patient with approximately 11 % of patients coming to the ED or being admitted to hospital (i.e. an estimated 88 % per year). We hypothesise that implementation of the SOS-HAE call centre management strategy should reduce this rate by 20 %, resulting in a rate of 68 % of ED visits or admissions per year over a 2-year period. If we consider the design effect due to cluster randomisation as relatively low (1.4), the estimated required sample size is 100 patients/arm for 85 % power and a 5 % alpha risk (two-sided comparison).

### Statistical analysis

Descriptive analyses will provide the following information for each continuous variable: mean value, standard deviation, 95 % confidence interval (CI), minimum, first quartile, median, third quartile and maximum and number of missing observations. Categorical variables will be expressed as numbers and percentages.

#### Analysis of primary outcome

The number of admissions for angioedema attacks will be analysed in the intent-to-treat population.

Because some patients may be blood relatives and because data from within the same family are not independent, the analysis will use generalised mixed models with the family included in the model as a random effect, the strategy as a fixed effect and with a binomial distribution of the variable of interest.

All tests will be two-sided.

#### Analysis of secondary outcomes

Secondary outcomes will be analysed using a mixed model ANOVA. The family will be introduced into the model as a random effect. All tests will be two-sided.

Number of admissions for a cause other than an angioedema attack per year for 2-year-period, mortality from an angioedema attack, mortality from another cause, number of ICU admissions per year, number of ED admissions per year, number of hospital stays, number of intubations per year, number of interventions by EMS, number of working days lost (and duration), costs (hospital and ambulatory costs) of patient care and SF-36 will all be estimated in each arm for 2 years.

## Discussion

Key priority issues in HAE management are reducing morbidity and mortality rates and socio-economic costs [2]. No study has ever been conducted to determine whether a central dedicated call centre for HAE attacks would reduce morbidity and the socio-economic impact of the disease.

The indications for the specific treatment of HAE attacks are well established. Plasma-derived C1INH and icatibant are the undisputed emergency drugs for life-threatening cases (laryngeal involvement), abdominal attacks and cases with facial involvement [2, 3, 20, 21]. Early home treatment is recommended [2, 15]. HAE patients trained in icatibant self-administration can recognise HAE attacks and decide when to inject [22], thereby avoiding hospital admission [12]. In an evaluation of the feasibility and efficacy of plasma-derived C1-INH in 31 patients with HAE, the time from attack onset to start of treatment was significantly shorter after than before training in self-injection [23]. Nevertheless, morbidity remains high in these patients. In one retrospective study, 73 patients (16 %) presented at the hospital ED with an attack and 59 % were admitted to hospital [13]. In another, 21 patients (11 %) presented at the hospital ED with an attack [11]. According to a recent prospective study, most of these patients visited the ED because they were short of medication or because they were unaware that emergency treatment could be self-administered. Our prospective controlled cluster-randomised trial would enable evaluation of the impact of a measure (dedicated call centre) that might shorten the time from attack onset to first contact with a healthcare professional.

HAE is a considerable economic burden both to the healthcare system and to HAE patients and their families [13]. Reducing the number of hospital admissions might help reduce this burden. We hypothesise that the use of a dedicated call centre providing expert advice for HAE might significantly reduce the number of admissions (morbidity rate) and thus the socio-economic costs associated with the disease.

### Trial status

Recruitment for the trial was completed in June 2014.

## Abbreviations

C1-INH: C1 inhibitor
ED: emergency department
EMS: emergency medical service
FXII-HAE: factor XII-HAE
HAE: hereditary angioedema
ICU: intensive care unit
SF-36: Short Form Health Survey
